# Structural Insights into the Evolution of a Non-Biological Protein: Importance of Surface Residues in Protein Fold Optimization

**DOI:** 10.1371/journal.pone.0000467

**Published:** 2007-05-23

**Authors:** Matthew D. Smith, Matthew A. Rosenow, Meitian Wang, James P. Allen, Jack W. Szostak, John C. Chaput

**Affiliations:** 1 Center for BioOptical Nanotechnology, The Biodesign Institute, Arizona State University, Tempe, Arizona, United States of America; 2 Department of Chemistry and Biochemistry, Arizona State University, Tempe, Arizona, United States of America; 3 Howard Hughes Medical Institute, Department of Molecular Biology, Massachusetts General Hospital, Boston, Massachusetts, United States of America; Institute of Molecular and Cell Biology, Singapore

## Abstract

Phylogenetic profiling of amino acid substitution patterns in proteins has led many to conclude that most structural information is carried by interior core residues that are solvent inaccessible. This conclusion is based on the observation that buried residues generally tolerate only conserved sequence changes, while surface residues allow more diverse chemical substitutions. This notion is now changing as it has become apparent that both core and surface residues play important roles in protein folding and stability. Unfortunately, the ability to identify specific mutations that will lead to enhanced stability remains a challenging problem. Here we discuss two mutations that emerged from an *in vitro* selection experiment designed to improve the folding stability of a non-biological ATP binding protein. These mutations alter two solvent accessible residues, and dramatically enhance the expression, solubility, thermal stability, and ligand binding affinity of the protein. The significance of both mutations was investigated individually and together, and the X-ray crystal structures of the parent sequence and double mutant protein were solved to a resolution limit of 2.8 and 1.65 Å, respectively. Comparative structural analysis of the evolved protein to proteins found in nature reveals that our non-biological protein evolved certain structural features shared by many thermophilic proteins. This experimental result suggests that protein fold optimization by *in vitro* selection offers a viable approach to generating stable variants of many naturally occurring proteins whose structures and functions are otherwise difficult to study.

## Introduction

We are interested in the extent to which nature samples the total structural diversity available in protein sequence space [1], [2]. In pursuit of finding novel proteins with properties similar to natural proteins, we have discovered that functional proteins can be selected from large unconstrained libraries of random amino acid sequences [1]. The non-biological proteins that emerge from these selections are discovered in much the same way that aptamers are selected from large pools of DNA and RNA [3]. We call this approach *de novo* protein evolution since the *in vitro* process of selection and amplification closely mimics the natural process of Darwinian evolution. In these experiments we explore how functional proteins evolve by imposing a selective pressure on a diverse population of unrelated sequences to enrich for molecules with a desired functional property. By starting from a random pool of proteins we attempt to sample broad regions of protein sequence space for different independent solutions to a given functional problem. Following several rounds of selection, we then recover the descendents of rare functional proteins that originated from starting libraries as large as 10^13^ different sequences. It is important to note that while our starting libraries are large by conventional standards, the total number of sequences analyzed represents an extremely sparse sampling of all possible sequence combinations, indicating that protein sequence space is surprisingly rich in functional diversity. In contrast to *de novo protein design*, which often relies on genetic algorithms to predict sequences consistent with a predetermined secondary or tertiary structure [4], [5], the process of *de novo protein evolution* requires no prior knowledge of a protein's structure or mechanism in order for a selection to be successful. As a result, larger regions of the protein universe can be explored for protein structures that are unanticipated and therefore potentially much more novel than structures predicted by design.

Initial experiments in *de novo* protein evolution began with an attempt to ascertain the frequency of ATP binding proteins in a sampling of all possible sequences in a contiguous library of 80 amino acids [1]. Here functional activity was viewed as the ability to bind a desired small molecule target with high affinity and specificity. Given that many protein domains have sequence lengths between 50 and 100 amino acids and ATP binding proteins are present in every major enzyme class, we felt that the random region and target choice were appropriate to identify small protein domains with simple ligand binding activity [6]. Because the probability of finding functional domains in a stochastic sampling of protein sequences was anticipated to be very low, perhaps too low to detect with conventional technologies, we used a cell-free selection system called messenger RNA display to construct a starting library of greater than 10^12^ non-redundant random-sequence proteins [7].

Following eight rounds of selection, four families of independent protein sequences were identified that bound ATP. One of these proteins (Family B) was optimized by directed evolution for improved binding affinity. DNA sequencing of the output from this selection revealed a distant clone (clone 18-19) that differed from the consensus sequence at 15 out of 80 positions and bound ATP with far greater affinity and specificity than all other clones from that round of selection [1]. The X-ray crystal structure for protein 18-19 was originally solved by Lo Surdo et al. and found to adopt a novel zinc-nucleated α/β-fold not yet observed by nature [8]. Structural comparison of protein 18-19 with the databank of biological protein folds revealed that our *de novo* evolved protein shared certain structural features with some proteins found in nature. These include a ligand binding site with stacking and electrostatic interactions similar to many natural nucleotide binding proteins and a zinc-binding site that is structurally analogous to the treble clef zinc binding motif [9], [10]. The discovery of close structural analogs between proteins that clearly do not share a common evolutionary ancestor suggests that certain structural motifs may be more common than previously thought, and may have emerged independently several times. How these motifs combine in three-dimensional space to form a stably folded protein might be less conserved.

Unlike many naturally occurring proteins, protein 18-19 requires high concentrations of free ligand in order to remain stably folded and soluble. In an attempt to overcome this limitation and to evolve a non-biological protein toward a folded state that more closely resembles the ligand-independent folded state of many natural proteins, we designed an *in vitro* selection experiment using mRNA display to isolate variants of protein 18-19 that remained bound to an ATP agarose affinity resin in the presence of increasing concentrations of chemical denaturant. Our goal was to examine the extent to which a *de novo* evolved protein, originally selected on the basis of ligand binding affinity, could be evolved to remain stably folded in the absence of exogenous ligand. We have previously used this strategy to identify variants of the Family B protein from a pool of sequences present in the output of the selection for improved binding affinity [2]. Here the pool was taken forward without mutagenesis to identify stable variants from within the existing population of molecules. While others have used phage display and ribosome display to improve the folding properties of natural proteins [11], [12], neither technique is capable of achieving both high library complexity and stable genotype-phenotype linkage under highly denaturing conditions. mRNA display, by contrast, is a cell-free selection system based on a covalent amide bond linking newly translated proteins to their encoding RNA message [13]. The absence of an *in vivo* transformation step allows mRNA display to avoid many of the intrinsic biases associated with cellular expression and to facilitate the construction of unusually large (>10^13^) protein libraries. We therefore reasoned that it might be possible to use mRNA-display to evolve a population of ATP binding proteins that remained stably folded in the absence of excess ligand if such variants existed and if our starting library contained enough sequence diversity to examine a significant region of sequence space around the ATP binding domain.

## Results

### Library Design and Construction

We began by generating a pool of protein 18-19 variants using error-prone PCR to produce random point mutations that would lead to single-residue changes in the protein [14]. Serial amplification was performed on the entire 240-nt gene-encoding region with a target mutagenesis rate of 3.5% per amino acid position. The quality of the pool was evaluated by sequencing 20 clones from the mutagenized DNA library. Analysis of the individual sequences revealed that the observed mutagenesis rate closely approximated the expected rate (3.7% versus 3.5% per amino acid position, respectively) with a roughly equal number of transitions and transversions. Two minor changes were made to the constant regions of the library relative to the starting library used to select protein 18-19. Two out-of-frame stop codons and a psoralen photo-crosslinking site were introduced downstream of the open reading frame to enhance fusion formation and reduce the time and cost of fusion construction.

**Figure 1:**
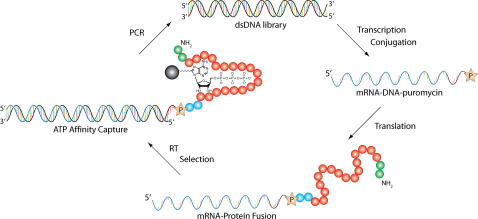
Evolutionary Optimization of Protein 18-19 by mRNA Display. The DNA library encodes randomly mutated variants of protein 18-19. For each round of selection the DNA library was transcribed, photoligated to a 3′-puromycin DNA linker, translated, and reverse transcribed to produce a pool of protein 18-19 variants displayed as mRNA-protein fusions. The library was incubated with ATP derivatized agarose in selection buffer supplemented with 1.5-3 M GuHCl, washed to remove the unbound material, and eluted with selection buffer containing 10 mM ATP. Eluted fractions were combined, amplified by PCR, and used as input into the next round of selection.

The DNA library was transcribed into RNA (Fig. 1) and photo-ligated to a DNA-puromycin linker using a psoralen photocrosslinking reagent to conjugate the synthetic puromycin linker to the 3′-end of the RNA. The pool of RNA-tagged DNA-puromycin templates was translated *in vitro* with rabbit reticulocyte lysate to produce a starting library of mRNA-protein fusions. The mRNA-protein fusions were then purified on oligo-dT cellulose and Ni-NTA agarose columns, and the RNA portion was reverse transcribed with DNA to prevent the unwanted enrichment of ATP binding RNA aptamers and to facilitate the amplification of selected proteins after separation on an ATP-agarose affinity column.

### Evolutionary Optimization of Protein 18-19 by mRNA Display

**Figure 2:**
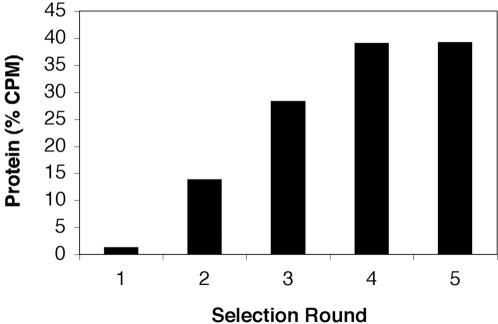
ATP Binding Selection Profile. The proportion of mRNA-protein fusions that bound to immobilized ATP and eluted with free ATP in selection buffer supplemented with 1.5 M GuHCl. The pool of protein variants used as the input into round 1 were generated by amplification of protein 18-19 under mutagenic conditions. The average mutagenic rate was 3.5% per amino acid position.

Evolutionary optimization of a protein fold requires developing a set of selection conditions that enable the structure to be optimized without compromising the function of the protein. Many examples of such trade-offs exist, especially among protein scaffolds designed to function as antibody mimics, where highly stable folds are reduced to marginally stable structures after evolving new functions [15]. We therefore chose to optimize protein 18-19 using a strategy that has both structural and functional constraints built into the selection step. Our approach to this problem involved enriching for well-folded variants by incubating the pool of mRNA-protein fusions with an ATP-derivatized affinity resin in the presence of guanidine hydrochloride (GuHCl). For each round of selection, the GuHCl concentration was gradually increased from 1.5 M to 3 M in an effort to ensure that less than 10% of the input to each round of selection was recovered in the elution step. By inactivating the pool by at least 90% prior to each round of selection and eluting under equilibrium conditions with 10 mM free ATP, we aimed to enrich for ATP binding proteins that remained folded and functional. Special care was taken to remove the guanidine hydrochloride prior to DNA amplification by PCR. Analysis of each of the selection steps in the starting denaturant concentration of 1.5 M GuHCl (Fig. 2) revealed that the fraction of library that bound to and eluted from the ATP agarose beads increased in each round, reaching ∼40% at round 4, but not increasing further at round 5. By contrast, protein 18-19 when constructed as an mRNA-fusion binds very poorly (∼1–2%) to ATP derivatized agarose when 1.5 M GuHCl is present in the binding, wash, and elution buffers.

### Sequence Analysis of Selected Proteins

We sequenced 53 clones from the output of round 5 to determine the sequence changes that led to the improved binding in the presence of GuHCl. Comparison of the selected sequences (Table 1a) showed that two amino acid substitutions dominated the selected population (N32D and D65V, present more than 35 times in 53 different sequences), while 11 other mutations had become selectively enriched (present more than 5 times in 53 different sequences). To examine this non-biological evolutionary history, we aligned (Table 1b) the primordial sequence from round 8 of the original selection from which all subsequent clones derive and the consensus sequence and highest affinity clone (protein 18-19) from round 18 of the binding optimized selection with the consensus sequence (protein DX) from the folding optimized selection performed on protein 18-19. These three sets of consensus sequences represent distinct stages in the evolutionary history of the Family B protein from its original discovery in a pool of 4 trillion random sequences to its optimization for improved binding and subsequent optimization for improved folding. Protein 18-19 was the highest affinity sequence from the binding optimized selection and the parent sequence for the current selection. 

Comparison of the different protein sequences revealed that one of the two mutations observed in the folding optimized protein (N32D) reverts an asparagine residue in protein 18-19 back to the aspartic acid residue present in the primordial sequence. The second mutation, D65V, was previously observed in a separate selection experiment designed to improve the folding stability of the Family B protein pool after optimization for improved binding affinity [2]. The consensus sequence from that selection (termed FOB, which stands for folding optimized protein from Family B) differs from protein DX at 17 out of 80 positions. The fact that protein 18-19 could achieve a similar degree of optimization with only two mutations seems to indicate that protein 18-19 was much closer to achieving structural optimization than other proteins from round 18 of the binding optimized selection. This is consistent with the previous observation that protein 18-19 exhibited greater ATP binding affinity than all other clones from that round of selection.

**Table 1:** Sequence Comparison of the In Vitro Selected Proteins.^a^

a	
Clone 18-19	MDYKDDDDKKTNWLKRIYRVRPCVKCKVAPRNWKVKNKHLRIYNMCKTCFNNSIDIGDDTYHGHDDWLMYADSKEISNT
Protein DX	-------------------------------D--------------------------------V--------------
Select mutations	-----------------F--G-----R----D-R----Y-------RA---YL-------H---V--------M-----
	1 10 20 30 40 50 60 70 80

b	
Primordial	MDYKDDDDKKTNWQKRIYRVKPCVICKVAPRDWWVENRHLRIYTMCKTCFSNCINYGDDTYYGHDDWLMYTDCKEFSNT
R18 Consensus	--------------------R------------R---------N--------S--------------------------
Protein 18-19	-------------L------R---K------N-K-K-K-----N------N-S-DI-----H--------A-S--I---
Protein DX	-------------L------R---K--------K-K-K-----N------N-S-DI-----H--V-----A-S--I---
	1 10 20 30 40 50 60 70 80

a Sequences of the Family B proteins from different points in the evolution history of this non-biological protein. Primordial and R18 consensus refer to the consensus sequences from rounds 8 and 18, respectively, in the original ATP-binding selection. Protein 18-19 was the highest affinity clone isolated from round 18. Protein DX is the double-mutant protein isolated by optimizing protein 18-19 for improved folding stability. The N32D mutation was present in 35 out 53 sequences. The D65V mutation was present in 45 out of 53 sequences. Other selected mutations were present ≥ 5 out of 53 sequences.

### Protein Expression and Purification

In order to investigation the functional significance of the two mutations observed in the folding optimized selection for protein 18-19, we cloned protein 18-19, protein 18-19 (N32D), protein 18-19 (D65V), and protein DX into a pMal plasmid and expressed the proteins in *E. coli* as N-terminal fusions of the maltose binding protein (MBP). Proteins were purified away from the crude lysate using an amylose affinity resin and quantified by UV spectroscopy. Comparison of the total amount of fusion protein obtained from a 1 L expression culture (Table 2) indicated that proteins containing one or both of the mutations expressed at higher levels than protein 18-19 (∼150 versus ∼65 mg/L culture, respectively). ATP binding proteins (ABP) were further purified by incubating the fusions with ATP agarose beads, proteolyzing the bound fusions with thrombin, washing away the MBP with buffer, and eluting the free ABP with phosphate buffer supplemented with 10 mM ATP. ATP binding proteins were then dialyzed with phosphate buffer to remove the excess ATP ligand. The two-step purification yielded highly pure ATP binding proteins suitable for biophysical characterization.

**Table 2:** Properties of Artificial Proteins.

Clone	M.W.^a^ (g/mol)	Expression (mg/L)	*K*_d_ (nM)	T_50_ (°C)
18-19	9696.9	88	1100 ± 78	57.5
N32D	9687.1	176	360 ± 12	77.1
D65 V	9683.6	131	640 ± 28	66.9
DX	9679.5	159	450 ± 36	79.5

a Molecular mass determined by maldi-tof mass spectrometry.

### Biophysical Characterization

We were interested in determining the extent to which our evolutionary optimization strategy led to protein variants with improved solubility and folding stability. We began by measuring the binding affinity (*K*_d_) of each protein for ATP by equilibrium spin-filtration [16]. Since it was difficult to determine how much ATP remained bound to the free protein after dialysis, *K*_d_ values were generated using MBP protein fusions. We have used this approach before to evaluate the binding affinity of other *de novo* evolved ATP binding proteins [1], [2]. Analysis of the results from this experiment (Table 2) revealed that the N32D mutation caused the greatest increase in ATP binding affinity, with a *K*_d_ of 360 nM, relative to protein 18-19, which binds ATP with a *K*_d_ of 1100 nM. Protein DX, which contains both the N32D and D65V mutations, binds ATP with a *K*_d_ of 450 nM and protein 18-19 containing the D65V mutation binds ATP with a *K*_d_ of 650 nM. Together, the selected mutations represent a ∼2-fold increase in ATP binding affinity over the parent sequence.

**Figure 3:**
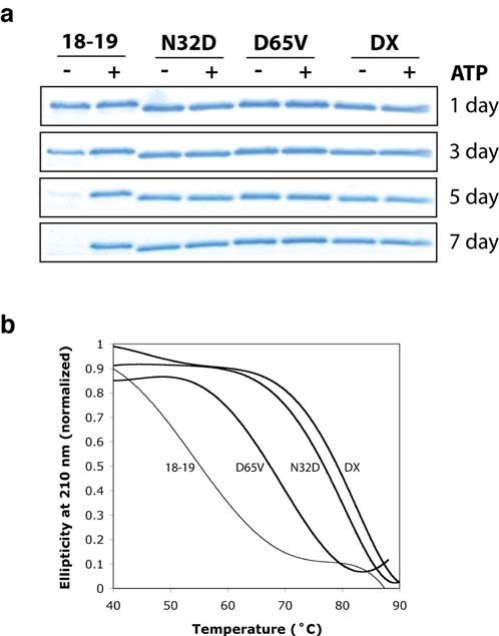
Characterization of the Selected Point Mutations. (A) ATP binding proteins 18-19, 18-19 (N32D), 18-19 (D65V), and DX were assayed for solubility in the absence and presence of free ATP. SDS-PAGE analysis shows that protein variants containing one or both of the selected mutations remain soluble in the absence of exogenous ATP. (B) Thermal denaturing curves for protein 18-19 and evolutionary optimized variants. The thermal stability of proteins 18-19, 18-19 (N32D), 18-19 (D65V), and DX were measured by temperature dependent circular dichroism at a fixed wavelength of 210 nm. Proteins containing one or both of the selected mutations exhibit higher relative thermal stability than the parent sequence.

To determine how selecting for improved folding stability correlated with improved protein solubility, we monitored the time-dependent precipitation of each protein in the absence and presence of ATP. Amylose purified MBP fusion proteins were separated into their respective proteins by thrombin proteolysis. After the proteolysis step, phenylmethylsulfonyl fluoride was added to the reaction mixture to prevent non-specific degradation of the ATP binding protein by thrombin. The reaction was then analyzed after 1, 3, 5, and 7 days by centrifuging the mixture to separate insoluble aggregates from soluble free protein, and analyzing the supernatant by SDS gel electrophoresis. Comparison of the individual proteins (Figure 3a) in the absence and presence of ATP indicated that all four proteins remain fully soluble after 7 days at room temperature when ATP is present in the reaction mixture. Experiments performed in the absence of ATP showed that protein 18-19 forms an insoluble precipitate after 3 days, while proteins containing one or both of the selected mutations remain soluble after 7 days. Analysis of the reaction mixture after 14 and 21 days at room temperature (data not shown) indicated that protein variants containing one or both of the selected mutations remained in the soluble fraction. The improved solubility observed for protein DX indicates that selecting for folding stability is a valid approach to isolating protein variants with improved solubility.

In order to determine the contribution of each mutation toward the improved folding stability of protein DX, we examined the stability of protein 18-19, protein 18-19 (N32D), protein 18-19 (D65V), and protein DX by thermal denaturation. The stability of the folded state was measured by monitoring changes in circular dichroism (210 nm) as a function of temperature. Denaturing profiles (Fig. 3b) for all four proteins were measured in the absence of exogenous ATP. All four proteins gave sigmoidal curves consistent with a single transition between the native and denatured states. We were able to determine the relative thermal stability of each protein by comparing the melting transition (T_50_), the temperature at which half of the protein is folded and half unfolded, for the parent protein and the two mutations individually and together. None of the proteins melted reversibly and as a result these values represent relative thermal stabilities rather than thermodynamic parameters. From this comparison, we found that protein DX exhibits significantly greater thermal stability than protein 18-19 (79.5°C versus 57.5°C, respectively). Of the two individual mutations, N32D appears to be responsible for most of the enhanced thermal stability, with a T_50_ of 77.1°C, while the D65V mutation is intermediate in stability and exhibits a T_50_ of 66.9°C.

### Structural Analysis

**Figure 4:**
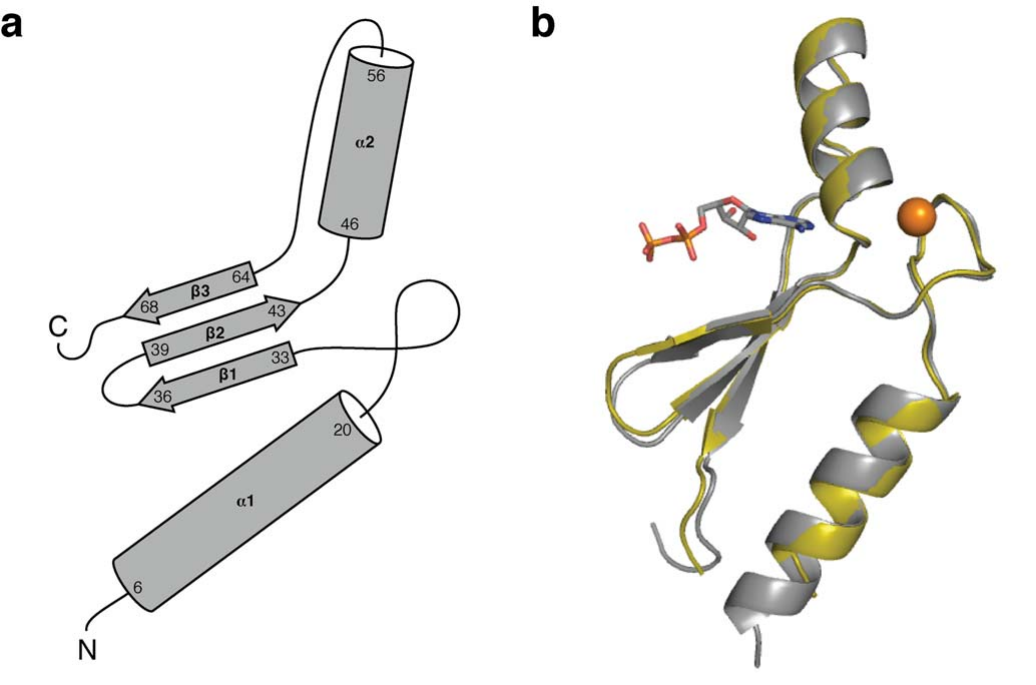
The X-ray Crystal Structure of Protein DX. (A) Schematic representation of the Family B scaffold. Mutations N32D and D65V transformed protein 8-19 into a stably folded water-soluble protein with significantly less ligand dependence than the parent sequence. (B) The three-dimensional structure (ribbon diagram) of protein DX (gray) superimposed with the parent sequence, protein 18-19 (gold). The zinc metal ion is shown in orange and the ATP ligand is colored by atom type.

We determined the X-ray crystal structures of protein 18-19 and the evolutionarily optimized variant, protein DX, to resolution limits of 2.8 Å and 1.65 Å, respectively (Table 3, Fig. S1). The structure of protein 18-19 was solved from the anomalous X-ray scattering of a zinc ion bound to the protein using multiwavelength anomalous dispersion (MAD) methods [17]. The structure for protein DX was initially solved by molecular replacement using the coordinates from protein 18-19, and later solved independently using single anomalous dispersion techniques. The Cα backbone trace of proteins 18-19 and DX can be superimposed (Fig. 4b) with a root mean square deviation (r.m.s.d.) of 0.51 Å from residues 7 to 71. Both structures closely approximate a previously determined X-ray crystal structure [8]. As illustrated in Figure 4a, the fold is characterized by an α-helix (residues 6–20), followed by an extended loop, then two short β-strands (residues 33–36 and 39–43), followed by a second α-helix (residues 46–56) that leads to another extended loop which terminates in a third β-strand (residues 64–68). The three β-strands form an antiparallel β-sheet that separates the two α-helices.

**Table 3:** Summary of Crystallographic Data.

	18-19			DX
*Space group*	*P*3_2_21			*P*3_2_21
*a* (Å)	71.46			72.79
*b* (Å)	71.46			72.79
*c* (Å)	55.26			54.75
*Data collection*				
Detector	ADSC Q315			RAXIS IV
Wavelength (Å)	1.2830	1.2834	1.2398	1.541
	peak	inflection	remote	
Resolution range (Å)	30-2.8	30-2.8	30-2.8	50-1.65
Total observations	19,167	16,744	17,881	102,431
Unique reflections	4,235	4,259	4,184	20,360
Redundancy a	4.3 (4.7)	3.9 (4.0)	4.4 (4.3)	5.0 (4.7)
Completeness (%)	100 (100)	99.9 (100)	99.9 (100)	99.1 (96.9)
<I>/<σ> a	6.6 (1.6)	4.4 (1.4)	4.2 (1.8)	17.4 (3.6)
*R*_sym_ a, b	0.099 (0.442)	0.139 (0.497)	0.137 (0.404)	0.056 (0.586)
*Model*				
R_work_	0.238			0.177
R_free_	0.296			0.195
Residues modeled	9-71			5-73
r.m.s.d. bond length (Å)	0.020			0.020
r.m.s.d. bond angle (°)	2.139			1.974
Average B factors (Å^2^)	35.1			24.6
all atoms				

a Values within parentheses refer to the highest resolution shell.

b *R*_sym_ = ∑*_h_* ∑*_i_* (|*I_i_*(*h*)-<*I*(*h*)>|)/∑*_h_* ∑*_i_ I_i_*(*h*), where *I_i_*(*h*) is the *i* ^th^ intensity measurement and <*I*(*h*)> is the weighted mean of all measurements of *I*(*h*).

**Figure 5:**
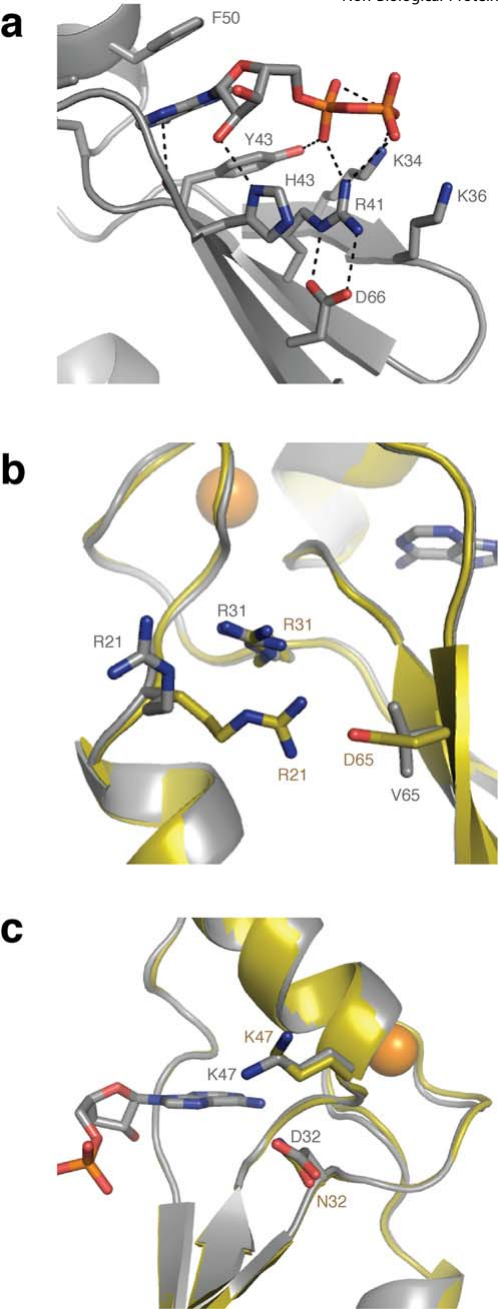
Structural Comparison of the Selected Mutations. (A) The region of the ATP binding site contains a network of pi-stacking (Y43 and F50) and electrostatic interactions (K34, K36, R41, and D66) that mediate ligand binding. (B) The D65V mutation removes a salt bridge on the hydrophobic side of the β-sheet and repositions several nearby loops and side-chains. (C) The N32D mutation caused a change in the electrostatic environment of the ligand binding site by forming an ion pair with K47.

**Figure 6:**
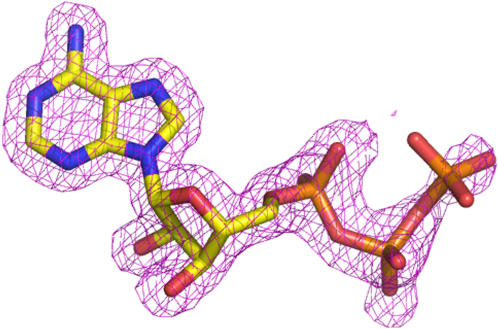
The crystal structure of the ATP ligand bound to the DX protein with a 2FoFc map contoured at a 1.5 sigma level. The electron density is much weaker at the gamma phosphate position indicating that this portion of the molecule is either disordered or has multiple conformations. The molecule is colored according to atom type.

The overall fold of the protein is stabilized by the presence of two cofactors. A zinc metal ion is bound by tetrahedral coordination to four invariant cysteine residues at positions 23, 26, 46, and 49. The identification of the metal ion as a divalent zinc atom was unambiguous based on the electron density maps generated from the multiple anomalous data. The presence of a zinc metal ion was not unexpected, and we have previously shown that protein 18-19 was a zinc-binding protein with the zinc metal required for folding [1]. In addition to the zinc ion, both structures also contain one ATP molecule bound to the protein in a well-defined nucleotide binding site. The adenine nucleobase is buried in a hydrophobic pocket (Fig. 5a) that is stabilized by stacking interactions involving the aromatic residues Tyr43 and Phe50 and hydrogen bonding interactions to mainchain carbonyl oxygens at residue positions Met45 and Gly63. The sugar moiety adopts a 2′-endo conformation with His64 hydrogen bonding with the 2′-hydroxyl group. The presence of several basic amino acid residues along the β-sheet, including Lys34, Lys36, and Arg41 provide a favorable electrostatic environment for the α-and β-phosphates, with the orientation of Arg41 controlled by a second shell of hydrogen bonding to Asp66. The ATP ligand is further stabilized by coordination to several additional bound water molecules. The γ-phosphate of ATP was less resolved (Fig. 6) in the electron density map. The large distance of 11 Å between the zinc atom and the center of the adenine nucleobase indicates that the zinc metal ion serves a structural role rather than functional role in ligand binding.

The Asp32 and Val65 amino acid mutations identified during the evolutionary optimization of protein 18-19 are located on opposite sides and faces of the β-sheet. The effects of the two mutations appear to be independent due to the long distance of 11.2 Å separates the alpha carbons of both residues. In protein 18-19, Asp65 is located on the hydrophobic face of the third strand and forms a salt bridge with Arg21, while Asn32 is located on the polar face of the first strand and contacts the purine base of ATP through a bridging water interaction. Although the overall fold of the protein does not change due to the mutations, several displacements of amino acid side chains and the loops are evident. The largest structural change occurs as a result of the D65V mutation (Fig. 5b), which eliminates the R21-D65 salt bridge, causing a significant repositioning of the Arg21 side chain. The change in the electrostatic interactions involving Arg21, Arg31, and Asp65 sidechains leads to a modest shift in the neighboring loops, with slight repositioning of the Arg31 sidechain. By comparison, the Asn→Asp mutation at position 32 (Figure 5c) does not alter the protein backbone, but instead results in an ion pair between Asp32 and adjacent Lys47, that is critical to balancing the dense positive charge present in the ATP ligand binding site. This change is evident when comparing the surface view of the electrostatic potential for both proteins (Fig. S2). Indeed a slight repositioning of the Lys47 sidechain toward the purine nucleobase can be seen in the crystal structure of protein DX. Small differences in the relative positioning of the ATP molecules in the two protein structures cannot be resolved due to the limiting resolution of the 18-19 structure.

## Discussion

Proteins are composed of amino acids whose sequence and identity determine the shape and function of the molecule. Unfortunately, very little is known about how such complex three-dimensional information is encoded in a one-dimensional amino acid sequence. Methods designed to reduce the complexity of this problem have relied heavily on substitution patterning to determine the relative tolerance of a particular amino acid position to change [18]–[20]. Two dominant approaches have been used to profile the substitution tolerance of proteins. The first involves a phylogenetic comparison of the sequences of evolutionary related proteins from different organisms [21]. Early work in this area showed that natural haemoglobin variants tolerated substitutions at exterior positions much more readily than interior positions [22], [23]. More recent comparisons made with larger data sets further support the initial finding that interior sites are more restrictive than exterior sites [24]. Given the large amount of sequence information currently available in databases of biological proteins, this method can be extremely effective at profiling amino acid positions determined by natural selection. The second approach relies on combinatorial methods, such as cassette mutagenesis, to identify the constraints on individual positions in a protein sequence [25]. The technique consists of a stepwise selection process whereby small sets of neighboring residues are randomized using degenerate DNA cassettes that encode all 20 naturally occurring amino acids, selected for function, and sequenced to identify acceptable substitutions. Repeating the process several times allows for complete coverage of specific regions of a protein domain or binding interface. Combinatorial approaches have been used many times to identify the structural determinants of proteins of both known and unknown structures [19]–[25]. Results from initial experiments on the DNA binding domain of λ repressor showed that core residues tend to have invariant or highly conserved side chains due to the precise nature of each residue within the jigsaw framework of the hydrophobic core, while surface residues tend to accept a wider-range of amino acid substitutions [25]. These and other studies have led to the general conclusion that amino acid residues that occupy solvent inaccessible positions in the hydrophobic core carry most of the information that defines the three-dimensional shape of the protein, while surface residues help maintain the stability of the fold [26]–[30].

In the present study, we used directed evolution to identify specific amino acid mutations from a pool of randomly mutated variants that enhance the stability of the protein fold. We then evaluated the mutations using biophysical methods to determine the effects of the mutations on the protein. The results from these experiments revealed that mutations N32D and D65V, located at solvent exposed positions, significantly enhance protein expression, solubility, thermal stability, and ligand binding affinity. This observation supports the notion that surface exposed residues carry more information that previously thought and make a significant contribution to protein folding and stability [27]–[30]. In evaluating the selected mutations, we wondered whether the stringent selection conditions used to evolve our non-biological protein led to the discovery of stabilization factors similar to those commonly attributed to thermophilic proteins [31]. Although the structural basis for how thermophilic and hyperthermophilic proteins achieve enhanced thermostability over their mesophilic homologues remains unclear, most studies suggest that extreme thermostability arises from a combination of factors that include increasing the content of polar charged residues at the expense of nonpolar sidechains, optimizing the number of electrostatic interactions, improving core packing, and reducing conformational entropy by minimizing large flexible loops [31], [32].

Structural comparison of our evolutionarily optimized protein to the parent structure (Fig. 5a–c) indicates that protein DX shares some of the same stabilizing factors as thermophilic proteins. The N32D mutation changes a polar uncharged residue to a polar charged residue, which improves the electrostatic environment of the protein by adding a negatively charged residue to a highly positively charged region of the protein. Balancing the charge density at this location was likely critical to stabilizing the protein in the absence of the ATP ligand. By comparison, the D65V mutation substitutes a polar charged residue with a non-polar residue on the hydrophobic side of the β-sheet. This mutation gives rise to a number of small structural changes, each of which appears to enhance the stability of the fold, as evident from the improved biophysical properties exhibited by the single-point mutations. The principle structural change involves the loss of the R21-D65 salt bridge (Fig. 5b), which leads to a change in the local electrostatic environment, causing the Arg21 to rotate away from the hydrophobic face of the β-sheet and reposition in the exposed solvent. The D65V substitution also causes a modest shift in nearby loops, which results in the optimization of electrostatic contacts at nearby residues. Together these smaller structural changes could reduce the conformational entropy of the protein by improving local packing and electrostatic interactions previously constrained by the R21-D65 salt bridge. Calculation of the solvent exposed surface area for each residue in the protein sequence indicated that positions 32 and 65 remained unaffected by the substitutions, reflecting the lack of any significant backbone changes. While further experiments are needed to define the precise nature of the N32D and D65V mutations, it is also possible that these residues may be involved in a complex network of long-range interactions that help modulate protein folding and ATP binding activity.

Although many unanswered questions remain about the amount of information that is necessary to encode a functional protein, other questions such as why these surface interactions were not identified in previous substitution experiments are easier to explain. The most likely reason why surface residues were initially dismissed in terms of information content resides in the fact that these interactions are often subtle and difficult to identify, and that the experimental procedures previously used to identify amino acid tolerance are often constrained by nature or experimental design. Both phylogenetic and combinatorial approaches are limited to the types and number of mutations found in the final sequence alignment. Here it is often difficult, without examining each substitution individually, to distinguish which mutations result from selection and which are caused by random drift, and the relative importance of each mutation with respect to fold and function [33]. Combinatorial approaches are further constrained by the selection conditions used to distinguish functionally active variants, and the window of residues assayed by each cassette [34]. In the original cassette mutagenesis studies, for example, an *in vivo* assay was used to identify functional variants of the λ repressor. While this assay was able to efficiently distinguish functional variants from nonfunctional variants, it was less effective at identifying variants that are both highly active and stably folded. By contrast, the *in vitro* selection strategy used to evolve protein 18-19 for improved folding stability required surviving members from each round of selection to be well folded and functional. A second difference between the *in vivo* approach used for cassette mutagenesis and the *in vitro* strategy taken here is that the *in vivo* assay focuses on only two or three residues at a time, making it difficult to identify the cumulative effect of multiple beneficial mutations. This is not to say that the individual mutations themselves could not be identified separately by colony screening methods, but rather that the iterative process of selection and amplification allowed us to converge on these mutations more rapidly. Overall, the high level of stringency and the potential to search a large library of broadly dispersed random point mutations are the two most likely reasons why we were able to identify subtle surface residues with important structural and functional properties.

The observation that solvent accessible positions carry significant amounts of structural and functional information suggests that it may be possible to optimize many naturally occurring proteins for improved solubility, folding stability, and functional activity. Here, we demonstrate that selecting for improved folding stability is a valid approach to improving the solubility and folding stability of a non-biological protein from random sequence origin. We suggest that this approach could be used to obtain well-folded soluble variants of many biologically relevant proteins whose structures and functions are otherwise difficult to study. The heterologous expression of many proteins in *E. coli* or yeast remains limited by the stability and solubility of the protein in the host system. In a particularly striking example, it has been noted that more than half of the genes in different bacterial species will require some form of optimization before soluble proteins can be obtained for crystallization [15]. Evolutionary optimization strategies that improve the stability of the protein without altering its backbone have significant value in many ongoing structure-function studies. Investigating how such changes enhance the stability of the fold will ultimately lead to a better understanding of how three-dimensional information is deposited in a one-dimensional amino acid sequence.

## Methods

### Construction of mRNA-Protein Fusion Library

Mutagenic PCR was used to generate the starting library with an average mutagenic rate of 3.5% per amino acid position based on DNA sequencing. The DNA library was amplified by PCR using the forward primer (5′-d-TTC TAA TAC GAC TCA CTA TAG GGT TTT TAT TTT TAA TTT TCT TTC AAA TAC TTC CAC CAT GGA CTA CAA AGA CGA CGA CGA T-3′) which contains the sequence information necessary for *in vitro* transcription and translation and the reverse primer (5′-d-ATA GCC GGT GCT ACC GCT CAG ACC CTT CGC AGA TCC AGA CAT TCC CAT ATG ATG-3′) that contains the information necessary to incorporate a unique three amino acid signature (AKG), two out-of-frame stop codons, and a psoralen photo-crosslinking site for ligation to the psoralen-DNA-puromycin oligonucleotide. The PCR product was phenol/chloroform extracted, ethanol precipitated, and transcribed with T7 RNA polymerase. The mRNA library was purified by denaturing PAGE, treated with RQ1 DNase (Promega), phenol/chloroform extracted, and concentrated by LiCl precipitation. Purified RNA was photochemically ligated to a psoralen-DNA-puromycin linker (5′-psoralen-TAGCCGGTG-(PEG_9_)_2_-dA_15_CC-puromycin; Glen Research, underlined positions denote 2′-methoxy nucleosides), by irradiating for 15 min at 366 nm in 96-well microtitler plate (50 ul per well). The crosslinked material was purified by denaturing PAGE and translated *in vitro* by incubating for 1 h at 30°C in 1 ml rabbit reticulocyte lysate (Nova Red, Novagen) and [^35^S]-methionine (Amersham Biosciences). In situ fusion formation was promoted by the addition of KCl and MgCl_2_ to final concentrations of 400 mM and 50 mM respectively, followed by a second incubation for 15 min at 25°C. mRNA-protein fusions were purified from the crude lysate by oligo (dT) and Ni-NTA chromatography (Qiagen). mRNA-protein fusions (100 pmol) were reverse transcribed with Superscript II RT (Gibco) using the RT-primer (5′-d-T_15_AA CCG CTC AGC TTG GCC TG-3′) to prevent RNA structure formation during the selection. Approximately 25 pmol of the starting library was used in the first round of selection.

### Selection of ATP-Binding Proteins

Displayed proteins (100 pmol) were incubated with C-8 linked ATP agarose beads (Sigma) in selection buffer [200 mM Hepes (pH 7.5), 4 mM MgCl_2_, 400 mM KCl, 1 mM Na_2_HPO_4_, 0.1% trition X-100, and 10 mM BME] supplemented with 1.5-3 M GuHCl for 2 h at 25°C in a 5 mL disposable column (Biorad). The column was drained, washed with 25 column volumes of selection buffer, and eluted stepwise over 10 min intervals with 0.25 ml aliquots of selection buffer supplemented with 10 mM ATP. Elution fractions were combined, NAP-5 (Pharmacia) exchanged into H_2_O, and PCR amplified. Following five rounds of selection, the amplified DNA was cloned into a TOPO vector (Invitrogen) and sequenced (MGH Core Facility).

### Protein Expression and Purification

Individual ATP binding proteins (ABP) were inserted into the pMal expression vector (pIADL14) using Bam HI and Hind III restriction sites. Plasmid DNA was transformed into one-shot chemically competent *E. coli* TOP10 cells (Invitrogen), cloned and sequenced (ASU Core Facility). Plasmid DNA was subcloned into *E. coli* BL21(DE3) strain (Invitrogen) for protein expression. Bacteria were grown in LB medium supplemented with 0.1 mM ZnSO_4_ at 37°C to an A_600_ of 0.8 and induced with 1 mM IPTG. Following an additional 4 h of growth, bacteria cells were harvested by centrifugation and re-suspended in 10 mL of phosphate buffer (25 mM NaH_2_PO_4_ (pH 8.0) and 250 mM NaCl). Cells were lysed by sonication in the presence of 1 mg/ml lysozyme. The crude lysate was clarified by filtration through a 0.45 µm PES filter (Millipore). MBP-fusion proteins were bound to an amylose column (New England Biolabs), washed with phosphate buffer and eluted with phosphate buffer supplemented with 10 mM maltose. MBP-fusion proteins were concentrated using an Amicon ultrafiltration device (Millipore). Protein concentration was determined by UV-spectroscopy at 280 nm using molar extinction coefficients calculated for each sequence. The yield of pure MBP-ABP fusion protein obtained from a 1 L culture of induced *E. coli* was ∼65–160 mg. Free ABP protein was obtained by thrombin proteolysis of the MBP-ABP fusion protein. MBP-ABP fusion proteins were bound to an ATP agarose column (Sigma) and cleaved with thrombin (Novagen). Free MBP was removed by washing with phosphate buffer and free ABP was eluted with phosphate buffer supplemented with 10 mM ATP. The eluted ABP was concentrated using a centricon filter (Millipore) and dialyzed against phosphate buffer. The yield of pure ABP obtained from a 1 L culture of induced E. coli was ∼15–30 mg.

### Thrombin Cleavage Assay for Protein Solubility

MBP-ABP fusion proteins (2 mg/mL) were incubated overnight at room temperature with thrombin (1 mg/mL, Sigma) to separate the MBP-ABP fusion into individual proteins. The relative amount of free ABP that remained in solution was monitored over time (1, 3, 5, and 7 days) by centrifuging the reaction mixture and analyzing a small aliquot (5 µL) of the supernatant on a 10–20% gradient SDS-PAGE (Tris-Glycine buffer system, BioRad). Comparing the band intensity of each mutant protein to the folding optimized protein provided a direct assessment of relative solubility.

### Determination of Equilibrium Dissociation Constants

Equilibrium dissociation constants (K_d_) for purified MBP-ABP fusion proteins were measured by equilibrium ultrafiltration. Apparent K_d_s were measured using trace γ-[^32^P]-ATP (Amersham Biosciences) and a series of concentrations of MBP-ABP fusion protein spanning the K_d app_. The data was iteratively fit (through nonlinear regression) using the computer program Deltagraph 4.0 (Red Rock Software) to the equation y = b+c((x/(x+K_d app_)), where y is the experimentally measured value (top counts-bottom counts/top counts), x is the protein concentration, b represents nonspecific binding to the protein and/or filter, and c is the maximum fraction of counts that can be bound.

### Circular Dichroism

Circular dichroism (CD) spectra were acquired using an Aviv CD Spectrometer Model 202. Spectra were recorded in phosphate buffer by monitoring the wavelength dependence of [*θ*] in 1 nm increments with a sampling time of 10 s. Melting curves were generated by monitoring the change in wavelength dependence of [*θ*] at 210 nm over a temperature gradient of 24–90°C. T_50_ values were determined by curve fitting using the program Deltagraph 4.0 (Red Rock Software).

### Protein Crystallization and X-ray diffraction measurements

The proteins were crystallized using the vapor diffusion technique. The initial conditions were based upon those described by Lo Surdo and coworkers with a large number of modifications [8]. The protein solution was prepared in 100 mM sodium phosphate pH 8.5 and 300 mM sodium chloride rather than 40 mM Tris HCl pH 7.5, 200 mM sodium chloride. In our protein solution, the 2 mM DTT reported by LoSurdo was not included but 10 mm ATP was present. The initial conditions yielded small crystals and after varying the conditions significantly larger crystals were produced. The optimal conditions for the 1–2 microliter protein drop are 20 mg/ml protein, 250 mM sodium citrate, 100 mM sodium phosphate pH 8.5, 10 mM ATP, 300 mM sodium chloride, and 0.4–0.8% x/v polyethylene glycol 400. The 1 ml reservoir contained 250 mM trisodium citrate and 22–25% w/v polyethylene glycol 400.

The crystals were frozen to 90 K and found to diffract to a resolution limit of 2.8 Å belonging to the space group *P*3_2_21. The structure of 18-19 was determined by use of multiple anomalous dispersion (MAD) phasing with diffraction data collected at the Advanced Light Source, Berkeley CA. A total of three data sets extending to a resolution limit of 2.8 Å were collected at the peak, edge, and remote wavelengths for Zn, 1.2830 Å, 1.2834 Å, and 1.2398 Å, respectively. The diffraction images were processed with MOSFLM [35] and SCALA [36]. The data are essentially fully complete to 2.8 Å and yielded excellent phases using SOLVE (figure of merit 0.40) and an initial model was automatically built using RESOLVE [37]. After iterations of model rebuilding using COOT [38] and refinement using REFMAC [39] the final structure has been refined with peak wavelength data to a *R_work_* and *R*_free_ of 0.238 and 0.296, respectively (Table 3). The refined model of 18-19 agrees within experimental error to the previously published structure by Lo Surdo and coworkers [8]. The protein structure is completely traced with the exception of the first eight residues forming the amino terminus and the last seven residues forming the carboxyl terminus region.

The DX protein structure was subsequently crystallized using the similar conditions. The crystals were frozen to 110 K and diffraction data was measured using a Rigaku R-AXIS IV^++^ image-plate area detector using Cu Kα radiation from a Rigaku RU-200HB rotating-anode X-ray generator (50 kV, 100 mA). The X-ray source was equipped with an Osmic confocal mirror assembly. The crystals belonged to the same space group as for the 18-19 protein with very similar cell constants but showed a significantly improved diffraction quality as they diffracted to a resolution limit of 1.65 Å. The diffraction data were integrated and scaled with HKL2000 [40]. The DX structure was solved by use of the 18-19 as the search model using the molecular replacement program MOLREP [41]. The results showed a single clear peak and the resulting model could be easily refined after substitution of the altered amino acid residues. The model rebuilding and refinement were carried out with COOT [38] and refinement using REFMAC [39]. The resulting model agrees with the electron density map as shown by the *R*_work_ and *R*_free_ of 0.177 and 0.195, respectively. An additional four residues (5 to 8) of the amino terminus region and two residues (72 to 73) of the carboxyl terminus region, which were not resolved in the 18-19 structure are now evident in the electron density maps and have been included in the DX model. The models were checked with PROCHECK and MolProbity [42], [43]. In addition, the DX1819 data contains weak but significant anomalous signals from one zinc ion, six sulfurs from cysteine and methionine residues, and one chloride anion which is located in the 2-fold axis at crystallographic dimmer interface. In fact, with known positions of these anomalous scatters, the DX1819 structure could be successfully phased with SAD phasing method [44] using SHARP [45] and SOLOMON [46]. This experimental map has an exceptional high quality, and is completely unbiased (some portions of the map are shown in (Figure S1). Therefore, this map was also used to validate the final model upon the completion of the refinement. All figures were prepared with Pym [47].

## Supporting Information

Figure S1 The three-dimensional structure of protein DX showing electron density at the zinc and ATP binding sites, and at residue positions D32 and V65. The electron density map at a 1 sigma level calculated using phases determined using SAD followed by SHARP analysis.
(9.77 MB TIF)

Figure S2 Surface view of proteins 18-19 and DX. A positive electrostatic patch evolved during the course of the ATP binding selection that led to improved ligand binding affinity for 18-19 (left). The large portion of positive electrostatic charge was later balanced with increased negative charge during the folding optimization selection that led to the evolution of protein DX (right). Positive, negative, and neutral regions are shown in blue, red, and white, respectively.
(7.42 MB TIF)
