# Supplementary figures and images for: Structural Insights into the Evolution of a Non-Biological Protein: Importance of Surface Residues in Protein Fold Optimization
Source: PLoS One. 2007 May 23;2(5):e467. doi: 10.1371/journal.pone.0000467 (PMC1867856; doi:10.1371/journal.pone.0000467)

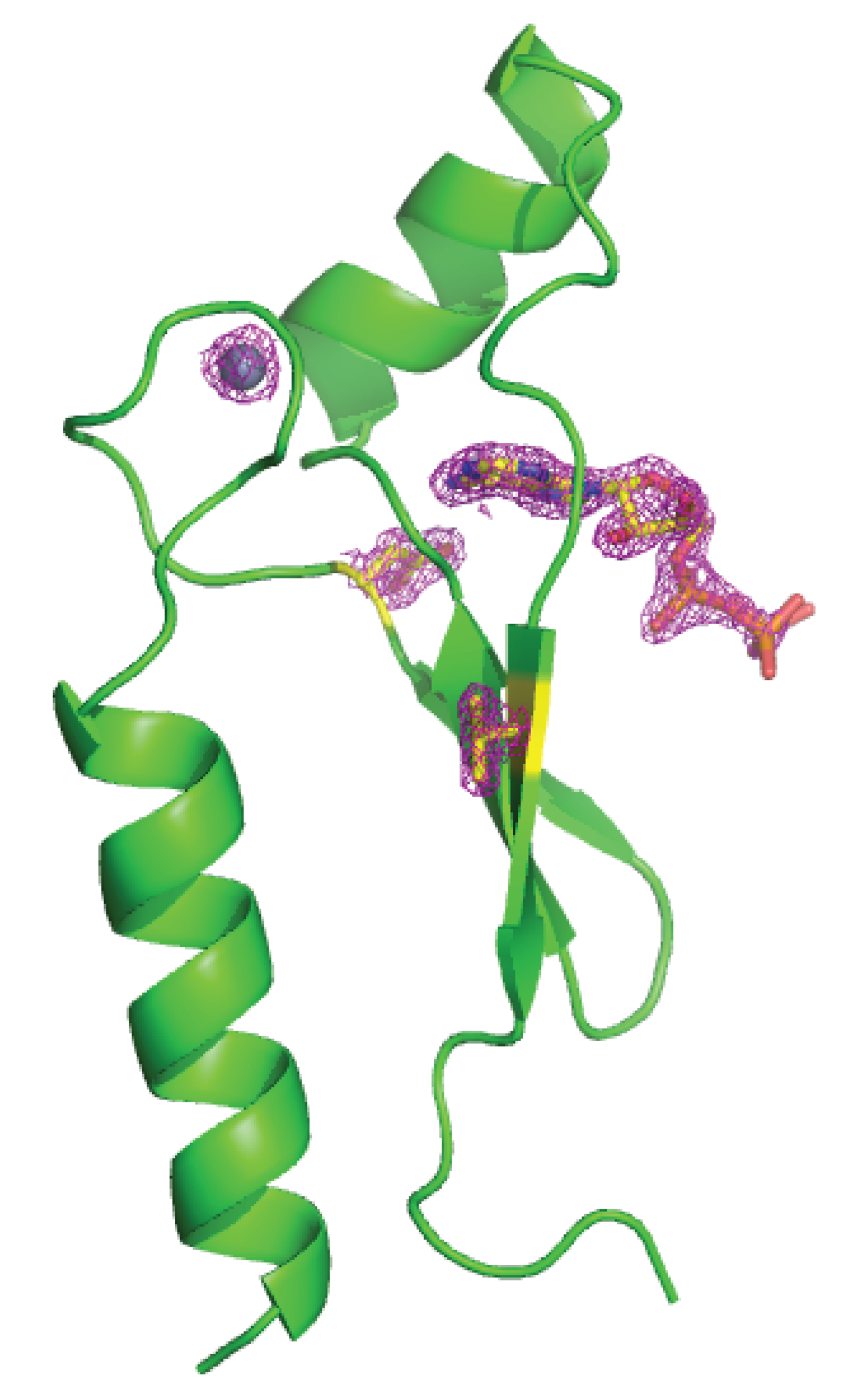

Supplement: Figure S1 — The three-dimensional structure of protein DX showing electron density at the zinc and ATP binding sites, and at residue positions D32 and V65. The electron density map at a 1 sigma level calculated using phases determined using SAD followed by SHARP analysis. (9.77 MB TIF) [file pone.0000467.s001.tif]

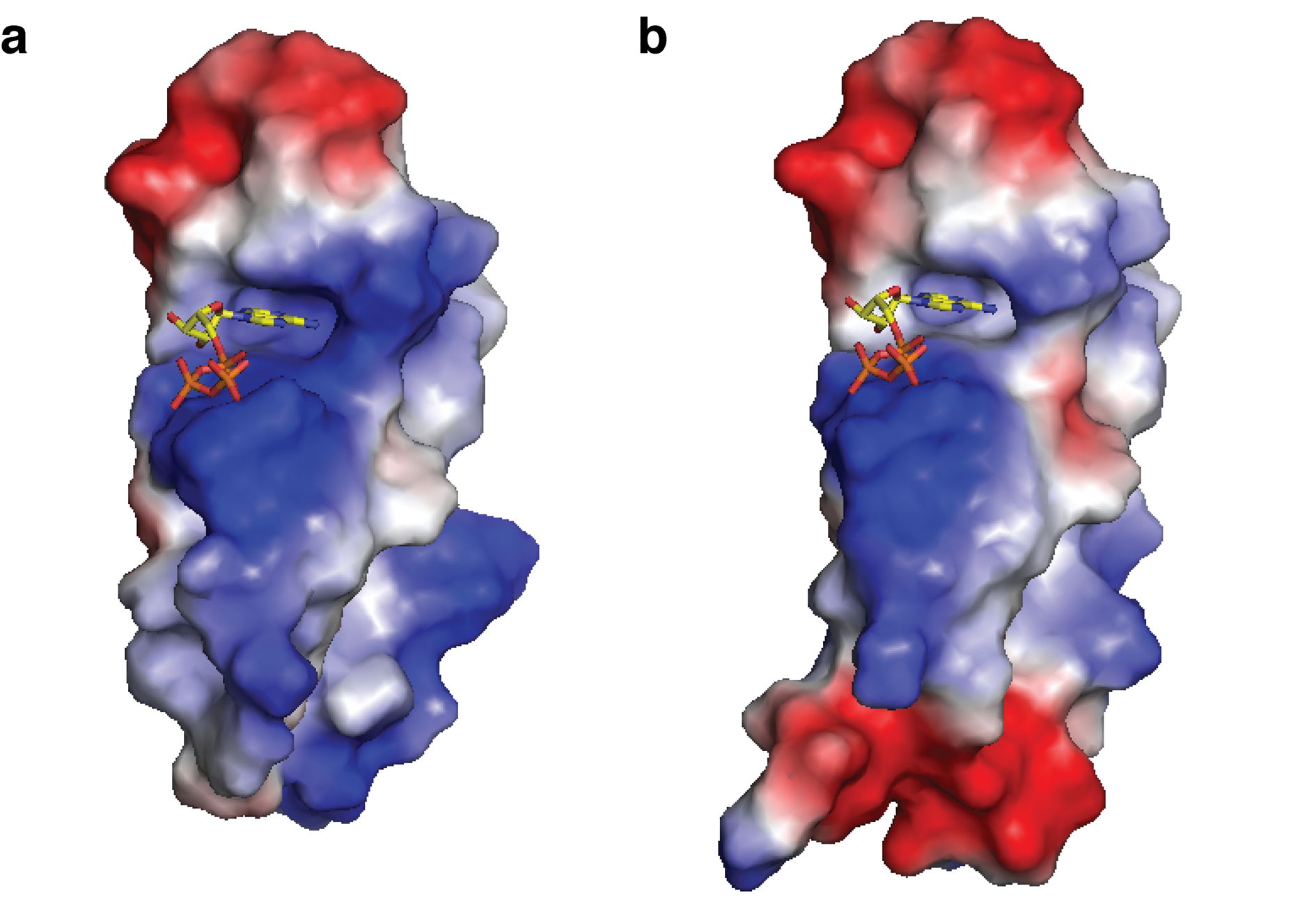

Supplement: Figure S2 — Surface view of proteins 18-19 and DX. A positive electrostatic patch evolved during the course of the ATP binding selection that led to improved ligand binding affinity for 18-19 (left). The large portion of positive electrostatic charge was later balanced with increased negative charge during the folding optimization selection that led to the evolution of protein DX (right). Positive, negative, and neutral regions are shown in blue, red, and white, respectively. (7.42 MB TIF) [file pone.0000467.s002.tif]
